# Graduating Nursing Students’ Empowerment and Related Factors: Comparative Study in Six European Countries

**DOI:** 10.3390/healthcare10050754

**Published:** 2022-04-19

**Authors:** Laura Visiers-Jiménez, Liisa Kuokkanen, Helena Leino-Kilpi, Eliisa Löyttyniemi, Riitta Turjamaa, Anna Brugnolli, Filomena Gaspar, Jana Nemcová, Alvisa Palese, Marília Rua, Renata Zelenikova, Satu Kajander-Unkuri

**Affiliations:** 1 Department of Nursing Science, Fundación San Juan de Dios, Centro de Ciencias de la Salud San Rafael, Universidad Antonio de Nebrija, 28036 Madrid, Spain; lvisiers@nebrija.es; 2 Department of Nursing Science, University of Turku, 20014 Turku, Finland; likuokka@gmail.com (L.K.); helena.leino-kilpi@utu.fi (H.L.-K.); 3 Turku University Hospital, 20521 Turku, Finland; 4 Department of Biostatistics, University of Turku, 20014 Turku, Finland; eliisa.loyttyniemi@utu.fi; 5 Department of Nursing Science, University of Eastern Finland, 70211 Kuopio, Finland; 6 School of Health Care, Savonia University of Applied Sciences, 70201 Kuopio, Finland; 7 Azienda per i Servizi Sanitari Provinciali, University of Verona, 38123 Trento, Italy; anna.brugnolli@apss.tn.it; 8 Department of Nursing Administration, Lisbon School of Nursing—ESEL (Escola Superior de Enfermagem de Lisboa), 1600-096 Lisbon, Portugal; mfgaspar@esel.pt; 9 Department of Nursing Science, Jessenius Faculty of Medicine in Martin, Comenius University in Bratislava, 03601 Martin, Slovakia; jana.nemcova@uniba.sk; 10 Department of Medicine, Udine University, 33100 Udine, Italy; alvisa.palese@uniud.it; 11 School of Health Sciences, University of Aveiro, 3810-193 Aveiro, Portugal; mrua@ua.pt; 12 Department of Nursing and Midwifery, Faculty of Medicine, University of Ostrava, 70103 Ostrava, Czech Republic; renata.zelenikova@osu.cz; 13 Diaconia University of Applied Sciences, 00580 Helsinki, Finland

**Keywords:** competence, empowerment, intention to leave, nursing education, nursing students

## Abstract

New nurses are needed in healthcare. To meet the role expectations of a registered nurse, nursing students must feel empowered at graduation. However, there are only a few studies focusing on nursing students’ empowerment. This study aims to describe and analyze graduating nursing students’ level of empowerment in six European countries and potential related factors. A comparative and cross-sectional study was performed in the Czech Republic, Finland, Italy, Portugal, Slovakia, and Spain with graduating nursing students (*n* = 1746) using the Essential Elements of Nurse Empowerment scale. Potentially related factors included age, gender, a previous degree in health care, work experience in health care, graduation to first-choice profession, intention to leave the nursing profession, level of study achievements, satisfaction with the current nursing programme, clinical practicums, theoretical education, and generic competence measured with the Nurse Competence Scale. The data were analysed statistically. Graduating nursing students’ self-assessed level of empowerment was moderate, with statistical differences between countries. Those with high empowerment had no intention to leave the nursing profession, had a higher level of study achievements, and a higher self-assessed generic competence level. The results suggest that empowerment needs to be enhanced during nursing education. Further research is needed to understand the development of empowerment during the early years of a nursing career.

## 1. Introduction

Nurses constitute the largest group of healthcare professionals and have high potential to influence population health [1]. Moreover, they have a key role in empowering patients regarding their own health [2] and in promoting the Sustainable Development Goals of the United Nations, such as good health and wellbeing [3]. However, a shortage of nurses globally [4,5] and the future challenges facing global healthcare, such as delivering health in conflict zones and dealing with the climate crisis and epidemics [4], have brought about an urgent need to invest in healthcare workers [4,5]. New nurses with a high competence are needed to respond to these challenges [1,6]. To be able to meet the role expectations of a registered nurse, nursing students must feel empowered already at graduation, in the very beginning phase of their nursing career [7]. However, there are only a few studies focusing on nursing students’ empowerment [8].

In Europe, new nurses’ intention to leave the nursing profession (hereafter “intention to leave”) seems to be a major concern for healthcare organizations. In a large European study involving 10 countries, for example, around 9.5% of practicing nurses reported intention to leave [9]. Moreover, as many as one in every four graduating nursing students (hereafter “GNSs”) in 10 European countries reported intention to leave, although they had not even graduated [10]. There are many reasons why new nurses have intention to leave [9,11]. The inconsistency between expectations and daily practice has been associated with the transition shock experienced by new nurses [12], causing stress and burnout [13] and intention to leave [9,11]. Furthermore, nurses’ intention to leave is caused by unsatisfactory workplace environments that have a harmful effect on the quality of nursing and patient safety [14]. Moreover, job satisfaction is recognized as a related factor of intention to leave and has been found to be connected to nurse empowerment [15,16]. 

Nurses’ empowerment can be described as a process as well as an outcome [17,18] and as an umbrella term for professional development at work [19], and its level varies over time [20]. In nursing, empowerment can be viewed through four theoretical approaches: organizational/structural theory; psychological theory; critical social theory [8,17]; and mixed theory, which combines the abovementioned theories [8]. Structural empowerment is significant for helping to deliver an effective workplace culture [21]. It focuses on elements such as nurses’ perception of empowerment and leadership, as well as on work outcomes, including engagement, job satisfaction, organizational commitment, and intention to leave [18,21]. Psychological empowerment focuses on an individual’s motivation in the workplace [22], acknowledging that empowerment can vary based on the context [18]. According to Spreitzer’s theory [22], four dimensions (competence, impact, meaning, and self-determination) must be present in order for empowerment to take place. The foundation of critical social theory is the premise that an individual has a capacity to act autonomously and is capable of self-reflection, emphasizing the idea of empowerment. Some groups have a subordinate status, which is sustained by social institutions and additional administrative units. In the nursing environment, these groups refer first of all to nurses and patients [8,17].

In this study, the theoretical framework consists of work empowerment as constructed by Kuokkanen and Leino-Kilpi [17], who developed their Ideal Model of Empowerment [23] utilizing the four cognitions of Thomas and Velthouse [24]. Later, the Essential Elements of Nurse Empowerment (EENE) scale was developed and tested based on the Ideal Model of Empowerment [23]. Work empowerment refers to work-related professional growth and development [19], and the EENE include ‘Qualities’, ‘Performance’, ‘Promote’, and ‘Impede’. For this study, ‘Qualities’ and ‘Performance’ were acquired as the conceptual framework. The element ‘Qualities’ refers to a “competent nurse who has personal power and is autonomous and personally responsible” and who is an “innovative, enthusiastic, promoter and forward-thinking”. The element ‘Performance’ refers to a nurse who “discusses openly, works for the common goal, and solves problems” [23] (p. 42).

The empowerment of nurses has been studied from different perspectives. In terms of qualities and performance, nurses’ empowerment level has been described, for example, as fairly positive [25], quite high [26], moderate [27], and rather high [28]. The empowerment of nurses has been found to relate to better patient care and overall job performance by nurses [29,30]; to increase patient-centred care [31]; to improve the clinical environment [8]; and to decrease the burnout, stress, and emotional exhaustion of nurses [29]. In addition, nurses who consider themselves empowered improve patient empowerment [8], use more effective work strategies and practices [29], and have a high commitment to their work organizations [15,21]. For newly graduated nurses (hereafter “NGNs”), the level of empowerment is described as fairly high [19], and empowerment is found to affect the transition phase positively [32] and to relate to job satisfaction and lower intentions to change jobs [19]. Additionally, a strong relationship between competence and empowerment has been found among NGNs [19,33]. 

Empowerment has previously been studied with respect to competence and other work-related factors, e.g., job satisfaction and intention to leave among NGNs. The strongest relationship has been detected between empowerment and competence, suggesting that higher generic competence corresponds to higher empowerment [19,33]. Competence refers to a nurse’s “functional adequacy and capacity to integrate knowledge and skills to attitudes and values into specific contextual situations of practice” [34] (pp. 330–331). Thus, the foundation of competence lies in attitudes, skills, and values in particular health care environments, while empowerment refers to self-improvement, self-belief, and self-efficacy [19]. Even though both empowerment and competence are essential concepts for GNSs’ professional development, according to our knowledge, no studies to date have investigated these elements together on a large scale. The target of this study was to fill in this gap. 

This study aimed to describe and analyze GNSs’ level of empowerment in six European countries and potential related factors. Three research questions were answered:

(1) What is the level of empowerment of GNSs?
(2) Are there differences between countries in the level of empowerment of GNSs?
(3) What background factors, if any, are related to the level of empowerment of GNSs?

## 2. Materials and Methods

### 2.1. Design

A comparative and cross-sectional survey design was applied in the study [35,36]. This study is a part of a European longitudinal study project, Competence of Nursing Students in Europe (COMPEUnurse), which focuses on the competence of nursing students and possible related factors at graduation and in the early years of practice in six European countries. Nursing students from six European countries (Czech Republic, Finland, Italy, Portugal, Slovakia, and Spain) participated in the study project at graduation by responding to several research instruments (https://sites.utu.fi/nursingscienceresearchprogrammes/pedagogic/, accessed on 26 March 2022). This study focuses on GNSs’ empowerment and related factors.

### 2.2. Participants

The target population included GNSs in six European countries located geographically across Europe. Voluntarily participating higher education institutions (HEIs) from the abovementioned countries were found via the Florence Network (https://theflorencenetwork.coventry.domains/about-the-florence-network/, accessed on 26 March 2022). Convenience sampling was used to enrol the students. The included GNSs were (1) studying in Bachelor of Nursing Science degree programs and (2) about to graduate. The sample size was estimated based on the Nurse Competence Scale [2], as the main instrument used in the COMPEUnurse research project; thus, the minimum sample size was 156 respondents/country (power = 0.80, significance level = 0.05 (two-tailed)), which was reached in every country. The participants’ (N = 4135; *n* = 1746) overall response rate was 42% (range 30–97% across the countries), which can be considered acceptable based on previous studies of the same topic [19,28] and a meta-analysis of response rates in online surveys [37].

### 2.3. Instruments

For measuring GNSs’ level of empowerment, elements ‘Qualities’ and ‘Performance’ of the Essential Elements of Nurse Empowerment (hereafter “EENE”) by Kuokkanen [23] were used, because these elements describe what empowered nurses are like and how they perform their tasks. The EENE was developed and tested from the original Ideal Model of Empowerment with logistic regression analysis and Lisrel analysis. The goodness of fit measures of the EENE were: Chi-Square = 2.03, df = 3, RMSEA = 0.00, and *p*-value = 0.566 [23].

In this study, the elements were used for the first time to assess how empowered the GNSs consider themselves. The element ‘Qualities’ includes two categories, expertise (6 items) and future orientedness (3 items), and the element ‘Performance’ includes the category of sociability (3 items). The GNSs responded to the items using a Visual Analogue Scale (VAS) from 0 to 100 (0 = does not apply to me in any case; 100 = it completely applies to me). To describe the level of empowerment, the VAS was divided into three parts: 0–50 for low, >50–75 for moderate, and >75–100 for a high level. The Cronbach’s alphas for the three categories varied between 0.69 and 0.86, and the total Cronbach’s alpha was 0.90, ranging from 0.88 to 0.91 across the countries.

Background factors potentially related to the level of empowerment in this study were age, gender, previous degree in health care, work experience in health care, graduation to 1st-choice profession, intention to leave, level of study achievements, satisfaction with current nursing programme, clinical practicums, and theoretical education. As research related to nursing students’ empowerment is scarce, these background factors are mainly based on the background factors that were investigated in students in the research project focusing on GNSs’ competence. In this study, we wanted to test the factors’ relationship with the level of empowerment. In addition, the Nurse Competence Scale (NCS) [38,39] was used to measure generic nursing competence and as an independent background factor, because a strong relationship between competence and empowerment has been found among NGNs [19,33]. The NCS was used to divide the GNSs into three groups based on their total VAS score for generic nursing competence (rather good: VAS mean < 50; good: VAS > 50–75; and very good: VAS > 75–100). The NCS consists of 73 items which form 7 competence categories. GNSs were asked to assess each competence item on a VAS scale (0–100) [38]. The NCS has shown evidence of validity and reliability in international studies with graduating [10] and recently graduated nursing students and more experienced nurses [39]. 

After obtaining the consent to use and translate the instruments from the copyright holders, a back-translation process [40] was conducted for the EENE into the languages needed (Czech, Finnish, Italian, Portuguese, Slovak, and Spanish). The NCS was back-translated into Czech, Portuguese, Slovak, and Spanish, as it already existed in Finnish and Italian. To ensure understandability and usability, the instruments were piloted in each country before the data collection. No changes were needed.

### 2.4. Data Collection

The data collection started in February 2018 and ended in September 2019 and was conducted with an electronic questionnaire. The national research teams in each country recruited the needed number of HEIs to achieve the sample size, and in these HEIs, all GNSs had an opportunity to participate in the study. Each participating HEI had a contact person(s) who sent the electronic questionnaire and an information letter to the potential GNSs by email during their final clinical practicum, and they answered anonymously. Moreover, two reminders were sent, two and four weeks after the first contact [35,36].

### 2.5. Ethics Approval and Consent to Participate

The European Code of Conduct for Research Integrity [41] and the ethical principles of the Declaration of Helsinki [42] were followed throughout the study. The ethical approval for the COMPEUnurse research project was obtained from the Ethics Committee of the University of Turku, Finland (Statement 16/2017, 6 March 2017). Students signed an informed consent when agreeing to participate in the study.

### 2.6. Statistical Analysis 

Continuous variables are presented with mean and standard deviations (SD). Statistical modelling for the EENE and subscores began with linear models (two-way analysis of variance or covariance), always including country in the model (univariate approach: age, gender, previous degree in health care, work experience in health care, graduation to 1st-choice profession, intention to leave, satisfaction with current nursing programme as a whole, satisfaction with clinical practicums, satisfaction with theoretical learning, level of study achievements, and NCS as categorised into three categories), and one-way analysis of variance for country only. First, the multivariable model included all significant explanatory variables from the univariate approach. Then, a multivariable linear model was further developed by dropping nonsignificant explanatory variables one by one. We also tested total NCS by country to see whether the association between competence and empowerment differed between the countries. From these models, adjusted means with 95% confidence intervals are presented for categorical variables and slope with 95% confidence interval for continuous covariates. The slope in the model describes whether the association between the continuous covariate and the EENE is positive or negative. Positive means that the covariate is high and the EENE is high as well, and when the covariate is low, the EENE is low as well. In addition, pairwise comparisons were corrected with Tukey’s method. The normal distribution assumption for this modelling was checked with studentized residuals. In addition, Pearson correlation coefficients were calculated. The association between categorized NCS and categorized empowerment was analysed with Fisher’s exact test. Intention to leave (fairly often and very often as combined; fairly seldom and never as combined) between the countries was compared using logistic regression. Cronbach’s alpha was calculated for EENE and subscores. In all analyses, if the subject had any missing values either in their responses or explanatory variables, the subject was automatically removed from the analyses, i.e., no imputation methods were performed. We considered *p*-values less than 0.05 (two-tailed) statistically significant. The statistical reporting was performed using SAS software, Version 9.4 of the SAS System for Windows (SAS Institute Inc., Cary, NC, USA).

## 3. Results

A total of 1746 GNSs responded to the study. However, the number of GNSs responding differed between the instruments used: the EENE, *n* = 1742; the NCS, *n* = 1716; and the background factors, *n* = 1746. The number of GNSs who responded to both the EENE and NCS was 1702. In all countries, the respondents were mostly female (range 81.8–98.1%), with a mean age ranging from 22.5 to 28.9 years (SD range 2.7–7.6). Overall, slightly over one third (range 3.7–69.2%) had a previous degree in health care, while two fifths (range 9.9–85.7%) had work experience in health care [12]. The number of GNSs who had fairly or very often had intention to leave varied between countries from 5.9% to 20.0%.

### 3.1. Level of Empowerment

The GNSs’ assessments of their level of empowerment ranged from 63.7 to 71.2 (mean 66.6, SD 14.8, Table 1), indicating a moderate level of empowerment (VAS > 50–75) across countries. GNSs’ assessments were the highest in the category of Expertise (VAS 68.7–72.2; mean 70.6, SD 15.1) and the lowest in the category of Sociability (VAS 46.2–62.7; mean 55.7, SD 21.6) (Table 1).

### 3.2. Differences between Countries in the Level of Empowerment of GNSs

The assessment differed between the countries. The level of empowerment was assessed to be the highest among GNSs in Spain (VAS mean 71.2) and the lowest in the Czech Republic and Slovakia (VAS mean 63.7). GNSs from the Czech Republic and Slovakia assessed their level of empowerment lower than those from Italy and Spain (*p* < 0.0001). At the category level, statistically significant differences were mostly seen in the category of Sociability, which was assessed as the lowest in all countries (Table 1).

### 3.3. The Association between Empowerment and Background Factors

In the linear model analysis, older (*p* = 0.021) and male (*p* = 0.0024) GNSs assessed their level of empowerment statistically significantly higher than other GNSs. In addition, GNSs who had never had intention to leave (*p* < 0.0001), who rated their study achievement level as excellent (*p* < 0.0001), and had very good generic competence (*p* < 0.0001) evaluated their empowerment level statistically significantly higher than others (Table 2).

Statistically significant differences occurred at the category level, too. GNSs who assessed their generic competence as very good assessed their level of empowerment higher in all categories compared to other GNSs (*p* < 0.0001) (Table 2).

GNSs’ intention to leave (fairly or very often) varied between countries from 5.9% to 20.0%, and statistically significant differences between the countries occurred (*p* < 0.0001). More specifically, Spanish and Italian GNSs had the lowest intention to leave (5.9% and 7.5%, respectively), and the differences were statistically significant between Portugal and Spain (*p* < 0.0001) and Italy and Portugal (*p* < 0.0001). There were also other statistically significant differences between countries (Table 3). The proportion of males and females differed between the countries (*p* < 0.0001), but intention to leave did not differ significantly between males and females (*p* = 0.94).

A statistically significant and positive correlation was seen between GNSs’ empowerment level and their generic competence (r = 0.64, *p* < 0.0001). In addition, a statistically significant and positive correlation was observed between each competence category and all three categories of empowerment. The correlations were strongest between Expertise and Work Role (r = 0.57, *p* < 0.0001) and Expertise and Managing Situations (r = 0.54, *p* < 0.0001) (Table 4).

We divided the GNSs into three groups based on their total VAS score for generic competence: (1) rather good level of competence, (2) good level of competence, and (3) very good level of competence. In terms of empowerment, the groups were: (1) low level (VAS mean < 50), (2) moderate level (VAS > 50–75), and (3) high level (VAS > 75–100). The largest group included GNSs (39.1%) who had a good level of generic competence (mean 62.8, SD 6.7) and a moderate level of empowerment (mean 63.3, SD 6.7). The second largest group included 16.6% of GNSs, who assessed their generic competence as being on a very high level (mean 83.5, SD 5.7) and their empowerment as being on a high level (mean 85.1, SD 6.3) (Table 5). A statistically significant association was seen between the groups of generic competence and the groups of empowerment (*p* < 0.0001).

## 4. Discussion

This study aimed to describe and analyze GNSs’ level of empowerment in six European countries based on their self-assessments and possible related factors. The study produced new knowledge from nursing students’ perspectives as to how empowered they consider themselves when entering the workforce as qualified nurses and contributes to our understanding of the beginning of a nursing career.

The main result of this study was that GNSs assessed their level of empowerment to be on a moderate level. On the VAS, ‘Moderate’ refers to scores around the middle or slightly higher. The comparison of our result with previous studies is difficult, as there are no studies of GNSs’ empowerment. In an earlier study, nursing students reported moderate degrees (mean 3.65; five-point Likert scale) of structural empowerment in their learning environment during nursing education [7]. NGNs have assessed their empowerment level in terms of qualities as fairly high (mean 3.95; five-point Likert scale) [19]. In addition, nurses’ empowerment level in terms of qualities and performance has been described as moderate (both means 4.19; five-point Likert scale) [27] and rather high (performance: mean 4.05; five-point Likert scale) [28]. These assessments represent, approximately, mean values over 70 on a VAS 0–100 scale. The GNSs’ assessments seem to be in line with these previous studies. 

The GNSs gave the highest assessments for ‘expertise’, which refers to a competent nurse with personal power, autonomy, and personal responsibility [23]. As qualified nurses must be able to work autonomously, make decisions independently, and bear a lot of personal responsibility [43], this is a favourable result. Future-orientedness, which refers to being an innovative, enthusiastic, and forward-thinking promoter [23], was assessed almost as high as ‘expertise’. This is a valuable result, as high assessments of ‘future-orientedness’ could lead to increasing innovativeness [19]. In a rapidly changing healthcare environment, future nurses must be able to identify and utilize innovations, such as innovative processes, products, and services, in order to enhance patient and population outcomes [44]. For example, nurse-led virtual clinics have been found to be patient-centred, cost-effective, and provide efficient delivery of care [45]. Based on the high assessments of ‘future-orientedness’, GNSs can work for the future and find solutions for problems, which is a good result from the viewpoint of dynamic healthcare. The lowest assessments were for ‘sociability’, which refers to a nurse who “discusses openly, works for the common goal, and solves problems” [23] (p. 42). The low assessments for ‘sociability’ are somewhat worrying, as at graduation, nursing students are required to solve complex and unpredictable problems [43]. This finding might be associated with the model of nursing education, which is based on the presence of one expert nurse supervisor in the clinical practicum, thus limiting the opportunity to discuss and be open to other members of the team [46]. Nurse managers are in a key role to promote an atmosphere of open discussion in their work units, and during clinical practicums it is important to engage nursing students as equals in the discussion within the unit. This could support students as they gain essential working life skills, such as listening to others, argumentation, and reflection with other members of the team.

We also found five background factors that were positively related to the level of empowerment. Age was a related factor, which was in line with a study among nurses [27] but contrary to a study among NGNs [19]. Moreover, a significant and strong positive correlation was detected between empowerment, including all its categories, and generic self-assessed competence, for all its categories. This finding is consistent with an earlier study among NGNs [33] and supports the suggestion that “competence and empowerment could be seen as a mutually supportive relationship” [33] (p. 453). The strongest positive correlation was between overall competence and ‘Expertise’, which is logical, as ‘Expertise’ refers to a competent nurse. At the category level, the strongest positive correlation was detected between the competence category ‘Work role’ and the empowerment category ‘Expertise’. All these results indicate that promoting competence development during nursing education is important from the viewpoint of students’ empowerment. 

Our results show that GNSs’ high empowerment level is clearly connected with a lower intention to leave. The number of GNSs and young nurses who had an intention to leave was considerably high in earlier studies [9,10]. Nurses’ intention to leave is a global phenomenon that gives rise to a growing shortage of nurses [1]. However, the knowledge of how many of these nurses with intention to leave actually leave the nursing profession is unclear [47]. Nevertheless, during a large shortage of nurses, it is worrying if even a small proportion of nurses leave their profession. Supporting new nurses’ empowerment could be one strategy to help them stay in the nursing profession. According to a previous systematic review and meta-analysis, empowered nurses are more highly committed to their work organizations [21]. In addition, NGNs who considered themselves to be empowered had no intention to leave [16,19] and were more satisfied with their jobs [22]. Thus, our results support these previous findings and suggest that enhancing empowerment early on during nursing education is important to increase commitment to the nursing profession. For example, a student-managed ward as a clinical practicum placement during the final year of nursing education can contribute to better preparation and more empowerment for the nursing role after graduation [48]. Moreover, in workplaces, nurse managers have an essential role to enhance supportive work environments and the professional development of new nurses and thus retain them by influencing their empowerment [19].

The level of study achievement was positively related to the level of empowerment. The results suggest that GNSs who assessed their study achievements as ‘excellent’ assessed their level of empowerment higher compared with other GNSs. NGNs have reported that self-confidence is gained by small experiences of achievement [32]. For a nursing student, excellent study achievements might be the factor that improves their self-confidence and leads to confidence in empowerment.

The assessments varied statistically significantly across countries. GNSs from Spain and Italy considered themselves as more empowered than GNSs from other countries. This could be explained by related background factors, such as generic competence and intention to leave. Generic competence correlated strongly with the level of empowerment. In a previous study, the self-assessed competence level of GNSs from Spain and Italy was higher than that of GNSs from the Czech Republic, Finland, and Slovakia [10]. It might be that GNSs with a lower level of generic competence do not consider themselves empowered. Moreover, GNSs from Italy and Spain reported a lower intention to leave compared with GNSs from other countries. Empowerment has been found to relate to higher job satisfaction and lower intentions to change jobs among NGNs [19], and empowered nurses are more highly committed to their work organizations [15,21]. According to a literature review [29], the link between empowerment and intention to leave has not been thoroughly investigated and, as far as we know, this was the first study to explore this association among GNSs. Nevertheless, these differences require further investigation. In addition, in the countries where the level of empowerment was lower, it is important to investigate whether this is a national trend and address strategies aimed at improving the empowerment among nursing students. 

The limitations of this study concern the sample. We used convenience sampling in each participating country. The sampling strategy and the fair response rate of 42% might influence the representativeness. Nevertheless, this is the first study to analyze and report comparisons of GNSs’ levels of empowerment in different European countries. The data collection period lasted 18 months, and no changes were made to the nursing curricula during the data collection period in the participating HEIs. Nevertheless, only cautious generalizations and preparatory conclusions can be made. However, our study provides guidelines for the design of future multicountry studies.

## 5. Conclusions

In the nursing role, it is important to feel empowered. There is a need for discussion regarding how to develop empowerment during nursing education. Promoting all areas of generic competence development during nursing education is important from the viewpoint of empowerment, and vice versa. Nursing students should be encouraged to take part in discussions within the unit during clinical practicums to enhance collegiality and problem-solving skills and to increase commitment to the nursing profession. There is also a need for discussion regarding how to support nursing students’ transition process to becoming qualified nurses and how to develop strategies focusing on preventing new nurses’ intention to leave. In addition, the results of this study are important for healthcare organizations, where nurse managers are in a key role to promote collegiality in their work units and nurses’ professional development and thus enhance nurse empowerment. In the end, an empowered and competent nurse will provide the safest and the most high-quality care for patients. Understanding the reasons for the differences between nursing students in different countries requires further research. Longitudinal research is needed on the development of the empowerment of new nurses during the early years of nursing practice.

## Figures and Tables

**Table 1:** GNSs’ level of empowerment analyzed with one-way ANOVA, pairwise comparison corrected with Tukey’s method (*n* = 1742).

Empowerment (EENE)					
Country	Expertise Mean (SD)	Future Orientedness Mean (SD)	Sociability Mean (SD)	Total Mean (SD)	Cronbach’s Alpha
Czech Republic (*n* = 212)	70.8 (14.2)	67.0 (16.1) ^1^	46.2 (20.6) ^1,3,6,7^	63.7 (14.0) ^1^	0.90
Finland (*n* = 336)	68.7 (14.8) ^9^	68.6 (15.9) ^1^	56.4 (19.1) ^4,9^	65.7 (13.6) ^4,5^	0.88
Italy (*n* = 334)	72.1 (15.2)	74.1 (16.9)	61.2 (22.5)	70.0 (15.2)	0.91
Portugal (*n* = 349)	71.5 (14.1)	66.6 (17.0) ^1^	54.2 (22.1) ^2,5^	66.0 (14.1) ^4,5^	0.90
Slovakia (*n* = 307)	69.0 (16.8)	63.3 (19.0) ^1,8^	52.6 (20.6)^1^	63.7 (15.7) ^1^	0.91
Spain (*n* = 204)	72.2 (15.3)	76.8 (16.5)	62.7 (20.7)	71.2 (14.4)	0.90
Overall	70.6 (15.1)	69.1 (17.5)	55.7 (21.6)	66.6 (14.8)	
Cronbach’s alpha	0.86	0.76	0.69	0.90	

Abbreviations: ANOVA, one-way analysis of variance; EENE, Essential Elements of Nurse Empowerment; GNSs, graduating nursing students; SD, standard deviation. Empowerment assessed with the Visual Analogue Scale 0–100. Superscripts 1–9 indicate statistically significant difference between this country and ^1^ Italy and Spain *p* < 0.0001, ^2^ Spain *p* < 0.0001, ^3^ Finland *p* < 0.0001, ^4^ Spain *p* < 0.01, ^5^ Italy *p* < 0.01, ^6^ Slovakia *p* < 0.01, ^7^ Portugal *p* < 0.01, ^8^ Finland *p* < 0.01, and ^9^ Italy *p* < 0.05.

**Table 2:** The association between GNSs’ background factors and their level of empowerment, analyzed with a linear model.

Empowerment (EENE)				
Background Factor	Expertise Adj Mean/ Slope B (95% CI) Adj *p* Value	Future Orientedness Adj Mean/ Slope B (95% CI) Adj *p* Value	Sociability Adj Mean/ Slope B (95% CI) Adj *p* Value	Total Adj Mean/ Slope B (95% CI) Adj *p* Value)
Age	0.13	0.15	0.096	0.13
	(0.0093–0.25)	(0.015–0.29)	(−0.085–0.28)	(0.020–0.24)
	0.035 *	0.030 *	0.3	0.021 *
Gender				
female	65.9 (62.1–69.7)	64.4 (60.0–68.8)	51.1 (45.4–56.8)	61.9 (58.4–65.4)
male	68.9 (64.8–73.0)	65.6 (60.9–70.3)	54.5 (48.4–60.7)	64.6 (60.8–68.4)
	0.0020 *	0.26	0.019 *	0.0024 *
Intention to leave				
never	70.3 (66.5–74.2)	72.6 (68.2–77.0)	56.3 (50.6–62.0)	67.5 (64.0–71.0)
fairly seldom	68.8 (65.0–72.7)	68.4 (64.0–72.7)	53.6 (47.9–59.2)	65.0 (61.5–68.5)
fairly often	66.6 (62.4–70.8)	62.5 (57.7–67.3)	52.8 (46.6–59.0)	62.4 (58.5–66.2)
very often	63.9 (58.6–69.2)	56.5 (50.5–62.5)	48.6 (40.8–56.5)	58.2 (53.4–63.1)
	0.0002 *	<0.0001 *	0.0042 *	<0.0001 *
Level of study achievements				
excellent	73.2 (71.1–75.3)	67.8 (65.4–70.1)	58.3 (55.2–61.3)	68.2 (66.3–70.1)
good	68.2 (66.8–69.6)	63.4 (61.8–65.1)	53.2 (51.0–55.3)	63.4 (62.1–64.7)
poor	64.7 (61.3–68.1)	62.1 (58.2–65.9)	49.3 (44.3–54.3)	60.3 (57.2–63.4)
very poor	63.5 (49.2–77.9)	66.7 (50.3–83.1)	50.6 (29.1–72.0)	61.2 (47.9–74.4)
	<0.0001 *	0.0004 *	0.0007 *	<0.0001 *
Competence (NCS)				
rather good (VAS 0–50)	55.0 (50.9–59.1)	52.7 (48.0–57.4)	39.7 (33.6–45.9)	50.7 (46.9–54.5)
good (VAS > 50–75)	67.8 (63.9–71.7)	65.3 (60.9–69.7)	52.5 (46.7–58.3)	63.5 (59.9–67.0)
very good (VAS > 75–100)	79.5 (75.5–83.5)	77.0 (72.5–81.6)	66.3 (60.3–72.2)	75.6 (72.0–79.3)
	<0.0001 *	<0.0001 *	<0.0001 *	<0.0001 *

Abbreviations: Adj mean, model-based mean; Adj *p*, *p* value; CI, confidence interval; EENE, Essential Elements of Nurse Empowerment; GNSs, graduating nursing students; NCS, Nurse Competence Scale; VAS, Visual Analogue Scale. Empowerment and competence were assessed with the Visual Analogue Scale 0–100. Adjusted means represent the model-based estimate for association between explanatory variable and EENE factors. In this linear multivariable model, all explanatory variables are in the same model. * Statistically significant *p*-value < 0.05. Bolded values are used for statistically significant values.

**Table 3:** Graduating nursing students’ intention to leave, analyzed with logistic regression (*n* = 1746).

Intention to Leave	Czech Republic (*n* = 212) *n* (%)	Finland (*n* = 331) *n* (%)	Italy (*n* = 335) *n* (%)	Portugal (*n* = 355) *n* (%)	Slovakia (*n* = 309) *n* (%)	Spain (*n* = 204) *n* (%)	Total (*n* = 1746) *n* (%)
fairly often/very often	39 (18.4) ^4,8^	50 (15.1) ^3,6^	25 (7.5)	71 (20.0) ^1,5,7^	37 (12.0) ^2^	12 (5.9)	234 (13.4)
fairly seldom/never	173 (81.6)	281 (84.9)	310 (92.5)	284 (80.0)	272 (88.0)	192 (94.1)	1512 (86.6)

Superscripts indicate statistically significant difference between this country and ^1^ Spain *p* < 0.0001, ^2^ Spain *p* = 0.024, ^3^ Spain *p* = 0.0018, ^4^ Spain and Italy *p* = 0.0002, ^5^ Italy *p* < 0.0001, ^6^ Italy *p* = 0.0022, ^7^ Slovakia *p* = 0.0056, and ^8^ Slovakia *p* = 0.043.

**Table 4:** Correlations between empowerment and generic competence (Pearson’s r) (*n* = 1702).

Empowerment (EENE)				
Generic Competence (NCS)	Expertise	Future Orientedness	Sociability	Total
Helping role	0.44 *	0.47 *	0.39 *	0.51 *
Teaching—coaching	0.51 *	0.49 *	0.41 *	0.56 *
Diagnostic functions	0.48 *	0.47 *	0.39 *	0.53 *
Managing situations	0.54 *	0.46 *	0.39 *	0.55 *
Therapeutic interventions	0.49 *	0.42 *	0.39 *	0.52 *
Ensuring quality	0.46 *	0.45 *	0.43 *	0.53 *
Work role	0.57 *	0.45 *	0.42 *	0.58 *
Overall competence	0.60 *	0.54 *	0.48 *	0.64 *

Abbreviations: EENE, Essential Elements of Nurse Empowerment; NCS, Nurse Competence Scale. * Significance level *p* < 0.0001.

**Table 5:** The association between empowerment and competence in different groups, analyzed with Fisher’s exact test (*n* = 1702).

Empowerment (EENE)				
Generic Competence (NCS)	Low (*n* = 231) VAS Mean (SD)	Moderate (*n* = 955) VAS Mean (SD)	High (*n* = 516) VAS Mean (SD)	*p*-Value
Rather good (*n* = 271)	*n* = 120/44%	*n* = 141/52%	*n* = 10/4%	
generic competence	38.7 (9.1)	42.2 (6.4)	44.2 (3.9)	
empowerment	40.4 (8.3)	59.5 (6.6)	79.0 (4.8)	
Good (*n* = 993)	*n* = 105/11%	*n* = 665/67%	*n* = 223/22%	
generic competence	59.0 (6.5)	62.8 (6.7)	66.1 (6.4)	<0.0001
empowerment	43.7 (7.0)	63.3 (6.7)	81.4 (5.2)	
Very good (*n* = 438)	*n* = 6/1%	*n* = 149/34%	*n* = 283/65%	
generic competence	80.9 (5.0)	80.9 (4.7)	83.5 (5.7)	
empowerment	47.4. (2.7)	67.5 (6.2)	85.1 (6.3)	

Abbreviations: EENE, Essential Elements of Nurse Empowerment; NCS, Nurse Competence Scale; SD, standard deviation; VAS, Visual Analogue Scale. Empowerment and competence were assessed with the Visual Analogue Scale 0–100. Generic competence: rather good—VAS mean < 50; good—VAS > 50–75; and very good—VAS > 75–100. Empowerment: low level—VAS mean < 50; moderate level—VAS > 50–75; and high level—VAS > 75–100.

## Data Availability

All data generated during this study are included in this manuscript.
